# Tapering and discontinuation of glucocorticoids in patients with rheumatoid arthritis treated with tofacitinib

**DOI:** 10.1038/s41598-023-42371-z

**Published:** 2023-09-20

**Authors:** Francesca Romana Spinelli, Cristina Garufi, Silvia Mancuso, Fulvia Ceccarelli, Simona Truglia, Fabrizio Conti

**Affiliations:** Dipartimento di Scienze Cliniche Internistiche, Anestesiologiche e Cardiovascolari – Rheumatology Unit, Sapienza Università di Roma, Rome, Italy

**Keywords:** Rheumatoid arthritis, Rheumatology

## Abstract

Although the rapid onset of effect of glucocorticoids (GCs) allows rapid control of rheumatoid arthritis (RA) symptoms, their chronic use may be associated with several adverse events. The 2022 update of EUALR recommendations for the management of patients with RA suggests to reduce and discontinue oral GCs as quickly as possible. Considering GCs as a "bridging therapy" to promptly reduce symptoms and control inflammation, fast-acting drugs such as tofacitinib could allow faster and safer tapering of GCs. The purpose of this pilot study was to evaluate the steroid-sparing effect of adding tofacitinib in patients with RA inadequately responsive to methotrexate taking concomitant GCs. In this open-label pilot study, we enrolled patients with moderate to severe RA on a stable dose of prednisone (5–12.5 mg/day) who started treatment with tofacitinib. After 1 month, in patients who achieved at least a moderate EULAR response (decrease of > 1.2 in DAS28_CRP), GCs was tapered according to a predetermined schedule until complete discontinuation at week 12. Disease activity was assessed after 4, 12, 24 and 48 weeks of treatment. The primary endpoint was the percentage of patients discontinuing GCs after 12 weeks of tofacitinib treatment. We enrolled 30 patients (26 F: 4 M, mean age 60 ± 13 years, mean disease duration 13.2 ± 7.8 years). The primary endpoint was achieved: 9 patients (30%) discontinued GCs at week-12. At week-24, other 12 patients (46%) withdrew GCs. The median prednisone dose decreased from 5 mg/day (interquartile range 5–10 mg) to 2.5 (0–5) mg/day at week 12 and 48 (p < 0.00001 vs baseline). At week 48, 12 out of 30 patients (40%) had discontinued prednisone. The percentage of patients achieving remission or low disease activity increased throughout the follow-up without any difference between patients who discontinued or not the GC. In this cohort of long-standing RA patients treated with tofacitinib, the discontinuation of glucocorticoids was achievable in up to 30% of patients. These results should encourage rheumatologists to consider GCs tapering and discontinuation of GCs, as suggested by the 2022 EULAR recommendations, an achievable goal.

## Introduction

The 2022 updated EULAR recommendations for the management of patients with Rheumatoid Arthritis (RA) address the issue of glucocorticoids (GCs) therapy stating that “*Short-term GCs should be considered when initiating or changing csDMARDs, in different dose regimens and routes of administration, but should be tapered and discontinued as rapidly as clinically feasible*”^1^. The EULAR task force suggests using GCs in combination with conventional synthetic Disease Modifying Anti Rheumatic Drugs (csDMARDs)—neglecting the combination of GCs with biological and synthetic targeted DMARDs—as "bridging therapy" until maximum effect is achieved^1^. Moreover, EULAR recommendation number 11 emphasizes that GCs should be discontinued if the patient is in sustained remission, before undertaking a dose reduction of DMARDs [biological (bDMARDs) or targeted synthetic (tsDMARDs)] and/or csDMARDs^1^.

The initial bridging add-on GCs therapy on a DMARD background would allow a rapid control of disease activity, while ensuring long-term structural benefits persisting even after GCs withdrawal^2^; in fact, doses ranging from 5 to 10 mg of prednisone per day seem to be associated to a significantly slower radiographic progression, lasting up to 2 years^3^.

Rheumatologists are aware of the potential toxicities associated to GCs but the “*dirty little secret”* of rheumatology is that prednisone is used often, and for periods of > 6 months”^4^. In clinical practice, however, the GCs discontinuation rate within 6 months after the initiation of a bDMARD have not significantly changed over time, despite the greater availability of advanced therapy and the prescription of GCs earlier in the course of the disease^5^. Moreover, the physician's attitude to prescribing GCs seems to predict long-term use: up to 45% of patients treated with bDMARDs or tsDMARDs for 6–9 months still take a median dose of prednisone of about 4 mg/day^6^. While GCs have a rapid effect on symptoms, chronic exposure to low to medium doses is associated with the occurrence of numerous adverse events such as osteoporosis, diabetes, hypertension, cardiovascular events and recurrent infections^7^. Nevertheless, most patients who have achieved a sustained remission after the introduction of a bDMARDs continue to take very low doses of prednisone^8^.

The Janus kinases (JAK) pathway is a common signaling pathway activated by many inflammatory cytokines, which has become a therapeutic target of RA treatment for the past decade. Tofacitinib is a potent inhibitor of JAK3 and JAK1 with a modest effect on JAK2. In addition to the clinical efficacy in RA patients showing inadequate response to methotrexate (MTX) or TNF-inhibitors^9–11^, tofacitinib in combination with background MTX also prevents progression of structural damage in one-year follow-up^12^. Significantly greater ACR responses were demonstrated for tofacitinib compared with placebo within the first 4 weeks of treatment, and ACR20 improvement was observed in all active treatment groups compared with placebo as early as week 1^10,13^.

About 60% of patients enrolled in randomized clinical trials with tofacitinib were co-treated with GC but little is known about tapering and the rate of GCs withdrawal during the treatment period^9,10,12^. If we consider using GCs as a “bridging therapy” in order to promptly reduce symptoms and control inflammation until the maximum effect of the DMARDs is reached, a rapid effect of this compound could allow for a faster and safer tapering of GC.

With this pilot study, we aimed to evaluate the steroid-sparing effect of adding tofacitinib in patients with RA inadequately responsive to MTX taking concomitant GC.

## Patients and methods

### Patients

Patients with diagnosis of RA according to 2010 ACR/EULAR classification criteria^14^, moderate-to-severe disease activity, inadequate responders to MTX and who are candidate to tofacitinib as per local indication, were eligible to the study. The main exclusion criteria were lack of informed consent acquisition, chronic use of non-steroidal anti-inflammatory drugs, other concomitant rheumatic diseases, pregnancy or pregnancy planning and contraindications to use either tofacitinib or glucocorticoids.

Patients under chronic GCs therapy (≥ 3 months), on prednisone dose ranging from 5 to 12.5 mg/day in a once-a-day morning administration, stable for at least 2 weeks before starting tofacitinib 5 mg twice/day (baseline visit) were enrolled. Prednisone was maintained at stable dose for the first 4 weeks after starting tofacitinib. Taking advantage of rapid action tofacitinib we set the first follow-up visit after 4 weeks: in patients achieving at least a moderate EULAR response (decrease of > 1.2 in CRP based Disease Activity Score 28 - DAS28_CRP), we started tapering GCs until complete discontinuation at week 12 according to a predetermined schedule based on baseline PDN dosage (Supplementary Table 1). We set the endpoint at 12 weeks as suggested by the 2016 EULAR recommendations which defined 3 months as a reasonable period within which to discontinue GCs^15^. All patients gave their written informed consent before being enrolled in the study.

### Study protocol

The project is a monocentric, prospective, open label, pilot study. Since no previous prospective studies has evaluated any GC-tapering schedule in RA patients treated with tofacitinib, the sample size of 30 patients was estimated assuming a 12-weeks response rate of 30% with a confidence interval of 90% and a margin of error of 15%.

The study protocol was approved by Sapienza Università di Roma – Policlinico Umberto I Ethical Committee (Protocol 674/18). All methods were carried out in accordance with relevant guidelines and regulations.

### Efficacy measures

The primary endpoint of the study was the percentage of patients discontinuing the PDN after 3 months since tofacitinib starting. Secondary endpoints were: 1) the percentage of patients requiring a new GCs course during the 12-months follow-up, 2) the percentage of patients in clinical remission or low disease activity at week 12, 24 and 48.

Demographic and clinical data—including disease duration, previous and current treatment, comorbidities and serology—was collected.

Disease activity and treatment response were assessed by DAS28_CRP, Clinical Disease Activity Index (CDAI) and Simplified Disease Activity Index (SDAI) at each visit—at baseline and after 4, 12, 24 and 48 weeks- by the same evaluator^16^.

### Safety assessment

Laboratory tests, complete physical examination, and other safety assessments were performed at each visit, with special attention to serious infections, Varicella Zoster virus reactivation, major cardiovascular adverse events (MACE), thromboembolic events, and neoplasms. Adverse events leading to temporary or permanent discontinuation of tofacitinib were also recorded.

### Statistical analysis

Patients’ characteristics were reported as number (percentage) for categorical variables and mean ± standard deviation (SD) or median (IQR) for continuous variables, according to the distribution. Single imputation using the Last Observation Carried Forward was used for missing data.

To identify variables associated with oral GCs decrease, patient baseline characteristics—including age, disease duration, number of tender and swollen joints, PGA, VAS pain, baseline GCs dose, number of previous bDMARDs—were compared between those who stopped the oral prednisone within 3 months and those who did not with logistic regression analysis.

Quantitative variables were compared with Wilcoxon and Mann–Whitney test for unpaired and paired non-parametric variables; qualitative variables were analyzed with chi-square test, or Fisher’s exact test, when appropriate. Change from baseline to week 12 for each outcome variable was compared between discontinuers and non-discontinuers using linear regression, adjusting for the baseline value of each outcome. For all statistical analyses, a p-value < 0.05 was considered statistically significant.

### Ethics approval and consent to participate

All enrolled subjects gave their written informed consent to participate in the study. The study protocol was approved by Sapienza Università di Roma – Policlinico Umberto I Ethical Committee (Protocol 674/18).

## Results

We enrolled 30 RA patients (26 F:4 M, mean age 60 ± 13, mean disease duration 13.2 ± 7.8 years). Table 1 summarizes the demographic and clinical data of the study population (Table 1).

**Table 1:** Demographic and baseline clinical data of the study population.

	Overall population (n = 30)	GCs discontinuers (n = 9)	GCs non discontinuers (n = 21)
F:M (% female)	26:4 (86.7)	8:1 (87.5)	18:3 (86.4)
Age, yrs (mean ± SD)	60 ± 13	54 ± 12	63 ± 13
Disease duration, yrs (mean + SD)	13.2 ± 7.8	10.4 ± 10.4	12.9 ± 7.4
N° previous bDMARDs [median (IQR)]	2 (2.5)	2 (2)	2 (2.5)
< 2 bDMARDs [n (%)]	12 (40)	3 (37.5)	9 (41)
bDMARD naïve [n (%)]	7 (23.3)	2 (25)	5 (22.7)
Baseline CDAI [median (IQR)]	30 (21.5)	28.7 (17.6)	31 (22)
Baseline SDAI [median (IQR)]	30.5 (23)	29.4 (17.5)	31.5 (24.3)
Baseline DAS28_CRP [median (IQR)]	5.0 (1.96)	5.3 (1.2)	5.1 (3)
Concomitant MTX [n (%)]	10 (33.3)	2 (22.2)	7 (33.3)
PDN dose, mg/day [median (IQR)]	5 (5)	5 (1.25)	5 (4.75)*
Daily PDN dose < 7.5 [n (%)]	20 (66.7)	6 (75)	14 (63.6)

*GCs* glucocorticoids, *Yrs* years, *bDMARDs* biological Disease Modifying Antirheumatic Drugs, *CDAI* clinical disease activity index, *SDAI* simplified disease activity index, *DAS28* disease activity score 28, *CRP* C reactive Protein, *MTX* methotrexate, *PDN* prednisone. *p < 0.00001

The study met the primary endpoint because 9 of 30 patients (30%) achieved complete discontinuation of prednisone at week 12. At 24 and 48 weeks, 12 out 30 (40%) had discontinued the daily prednisone. Although they had achieved at least a moderate EULAR response, 10 patients did not discontinue at week 12; multivariate analysis showed that factors negatively associated with GCs withdrawal after 12 weeks were age (p = 0.014), disease duration (p = 0.045), and baseline PGA (p = 0.028). Of the 10 patients who did not discontinue GCs at week 12 despite a moderate EULAR response, 4 patients discontinued by the end of 12-monbths follow-up. After 48 weeks, baseline negative predicting factors for discontinuation were age (p = 0.022) and disease duration (p = 0.025).

**Figure 1:**
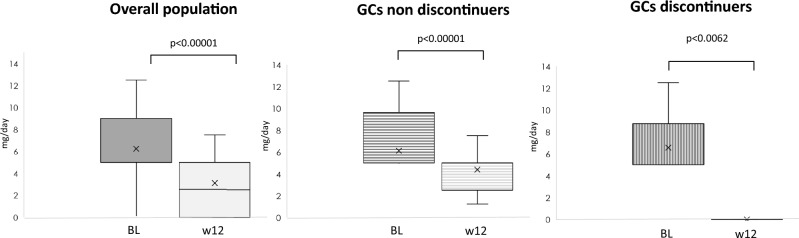
Tapering of glucocorticoids in the overall population, patients who did not discontinued and in those who discontinued the daily prednisone.

**Figure 2:**
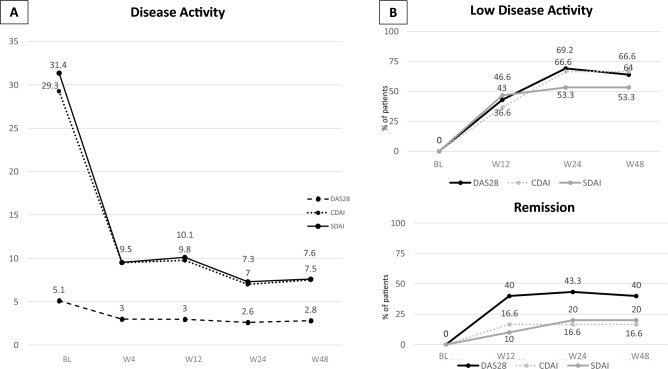
Disease activity (**A**) and treatment target achievement (**B**) during the follow-up in the overall population. *DAS28* disease activity score 28, *CDAI* clinical disease activity index, *SDAI* simplified disease activity index. *BL* baseline, *W4* week 4, *W12* week 12, *W24* week 24, *W48* week 48.

**Figure 3:**
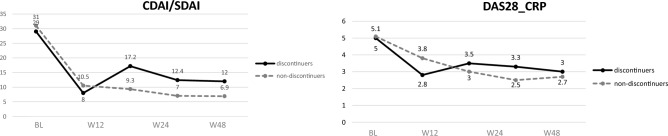
Disease activity during the follow-up glucocorticoids discontinuers and non-discontinuers. *DAS28* disease activity score 28, *CDAI* clinical disease activity index, *SDAI* simplified disease activity index. *BL* baseline, *W4* week 4, *W12* week 12, *W24* week 24, *W48* week 48.

Overall, the median baseline prednisone dose was 5 mg/day (IQR 5–10 mg); after 12 weeks the median dose decreased to 2.5 (0–5) mg/day (p < 0.00001). Nine patients further reduce the GCs dose from week 12 to week 48 [from 5 (5–7.5) mg to 1.25 (0–2.5) mg] (p < 0.00001). Seven patients continued a stable dose of prednisone of 5 (4.37–5.5) mg. During the follow up 4 patients had to increase the dose of GCs from 1.25 (1.25–2.5) mg to 5 mg; one more patient had to reintroduce 5 mg of prednisone after stopping at week 12. Figure 1 shows the reduction of GCs during the follow-up in the whole cohort, in GCs-discontinuers and non-discontinuers at week 12 (Fig. 1). Disease activity significantly decreased already after 4 weeks of treatment in the overall population, without difference between patients who discontinued GCs and those who did not (Figs. 2A and 3).

The percentage of patients achieving the low disease activity and remission according to DAS28_CRP, CDAI and SDAI is reported in Fig. 2B. We observed no difference in the percentage of patients who reached the goal among patients who discontinued or not discontinued GCs.

During the 48 weeks of the study, 3 patients discontinued tofacitinib for adverse events (one for the onset of endometrial malignancy after 5 months of treatment, one for serious infection, and one for paresthesia). As for the adverse events of special interest, none of the patients reported Varicella Zoster reactivation and no case of MACE nor thromboembolic events were recorded.

## Discussion

This study was designed to evaluate the steroid-sparing effect of tofacitinib in patients with RA. To the best of our knowledge, this is the first prospective study that evaluated the feasibility of a predetermined GCs discontinuation schedule in patients starting treatment with tofacitinib and showed that as early as 12 weeks after the initiation of treatment, 30% of patients were able to discontinue their daily GCs dose. The most recent recommendations for RA management suggest reducing the dosage of GCs as soon as possible until discontinuation^1^. In fact, the dose and duration of steroid therapy affect the safety profile and there is no agreement on the definition of the “safe” dose^17^. The optimal dose and duration of GCs therapy and the best strategy to taper and withdraw GCs are still scarce. A recent systematic literature review reported 14 different regimens proposed to reduce and/or stop oral GCs^18^. Randomized clinical trials on RA drugs provide inadequate information on GC therapy during treatment with biologicals and do not allow definitive conclusions to be drown on GC discontinuation, considering also that the steroid-sparing effect never appears as a secondary endpoint^19^.

A sub-analysis of the ACTION (AbataCepT In rOutiNe clinical practice) study showed that over a 24-month period from the start of abatacept, about 40% of the 734 patients taking GCs at the beginning of the study were able to reduce their prednisone dose, mostly within the first 3 months of treatment, and that the median GCs dose decreased from 7.5 mg/day to 5 mg/day after 24 months of treatment^20^. The need to evaluate the steroid-sparing effect of RA drugs is genuine and relevant, as shown by real-life studies. Most of the evidence in real-world settings comes from retrospective studies evaluating the effect of bDMARDs on GCs therapy in patients with long-standing RA such as those enrolled in our study. Many years ago, Naumann et al. described GCs dose reduction concomitant with decreased disease activity as early as 3 months after initiation of a TNF inhibitor in 87 patients with RA; during a 5-year follow-up, 81% of patients reduced GCs dose and 32% of patients discontinued therapy^21^.

In a French, retrospective study published in 2009, the authors observed an overall 30% reduction in oral GCs dose in the first year of treatment with TNF inhibitors, starting already after 3 months; on the other hand, 61% of patients were still on low-dose prednisone after 1 year^7^. More recently, the analysis of the TReasure database showed that after a median of 59 months, 28.4% of the 1936 patients who received GCs at the registry entry stopped the concomitant steroid^22^. The retrospective nature of the study explains the lack of prespecified criteria for the GCs tapering. The SPARE-1 study included RA patients treated with tocilizumab (TCZ) and an oral prednisone dose > 5 mg/day and aimed to evaluate the proportion of patients who reduced their GCs dose below 5 mg/day after 12 months of TCZ^22^. At the end of the follow-up, 40% of patients achieved the target dose; RA duration no longer than 5 years, daily prednisone dose < 7.5 mg and low baseline ESR were predictive of PDN reduction below 5 mg/day^23^.

Fernandez-Nebro et al. prospectively followed-up for 24 months 161 patients who started infliximab, etanercept or adalimumab, showing that nearly 60% of patients who were taking GCs at baseline was able to discontinue and the remaining patients significantly reduced the daily dose^24^. In a previous paper published by our group, we demonstrated a 56% reduction in the percentage of RA patients treated with baricitinib who were taking glucocorticoids after 24 weeks; in addition, we recorded a significant reduction in the daily dose of prednisone from a median of 5 mg/day to 0 mg/day, concomitant with a significant reduction in pain as early as 4 weeks^25^. Similarly, in the present study, the fast effect of tofacitinib on pain and disease activity certainly contributed to the fast reduction of the GCs dose, until complete withdrawal; indeed, about one third of the enrolled patients stopped the daily GCs within 12 weeks from tofacitinib initiation.

The initial bridging add-on GCs therapy on a DMARD background would allow a rapid control of disease activity which also ensures long-term structural benefits, persisting even after GCs withdrawal; doses ranging from 5 to 10 of prednisone per day seem to be associated to a significantly slower radiographic progression^2^. A post-hoc analysis of 6 phase III randomized controlled trials with tofacitinib suggested that concomitant use of GCs did not affect both clinical and radiographic efficacy of the drug^26^. Prospectively evaluating the efficacy of tofacitinib according to GCs discontinuation, we observed no difference in the reduction of disease activity as assessed by DAS28_CRP, CDAI, and SDAI; moreover, low disease activity or remission was achieved regardless the concomitant use of GCs. In contrast, data from a Turkish registry showed a significantly higher DAS28_CRP score and a significantly lower percentage of patients achieving low disease activity or remission in patients continuing with GCs concomitant with bDMARDs or tsDMARDs^20^. Data on the effect of GCs on clinical response to TNF inhibitors are also discordant and have shown a higher or lower percentage of patients reaching the treatment goal^27,28^. In the SEMIRA trial (The Steroid EliMination In Rheumatoid Arthritis) patients were randomly allocated to the continued-prednisone or tapered-prednisone regimen; two-thirds of patients who achieved the low disease activity with tocilizumab tapered the steroid dose in the 24-week study but, unlike what we have observed in our patients, patients who continued prednisone had a better control of the disease activity^29^.

In our study, age at enrolment and disease duration were negatively associated to the GCs discontinuation. This is not surprising, since the results of a recent double-blind, placebo-controlled study showed that elderly patients (older than 70 years at baseline) with established and severe RA had beneficial effects in the long term, both in terms of disease activity and joint damage, from taking low-dose prednisolone in addition to baseline therapy^30^. However, this study did not include patients taking tsDMARDs. Recently, data from the Veterans Affairs Rheumatoid Arthritis registry showed that 54% of patients tapered and 33% discontinued the oral GCs; younger age, positive Rheumatoid Factor, higher ESR at enrolment, a greater number of previous csDMARDs and higher average glucocorticoid dose over the 30 days before the index date were all significantly associated to GC tapering and discontinuation^31^.

The 2013 EULAR recommendations on the management of medium to high-dose glucocorticoids in rheumatic disease state that “*when it is decided to start glucocorticoid treatment, comorbidities and risk factors for adverse effects should be evaluated and treated where indicated *[…]”^32^. Especially at medium–high doses, GCs are commonly associated to the occurrence of undesirable effects including osteoporosis, diabetes, hypertension, cardiovascular events and recurrent infections. The sparing of GCs—as emerged from our results—may be beneficial in terms of short-term safety (infectious risk, in particular herpes Zoster reactivation) and long-term safety (metabolic and cardiovascular events as well as osteoporotic fracture). A large study on RA patients from the CorEvitas registry showed that already after 6 months of treatment, at a daily dose greater than 5 mg, GCs increased the risk of cardiovascular events^33^.

Some of these adverse effects—mainly infections and cardio-metabolic effects raise concerns in patients treated both with bDMARDs and tsDMARDs; in particular, the results of the ORAL Surveillance study raised the warning about cardiovascular safety in patients treated with tofacitinib^34^.

The present study has some limitations. The small sample size did not allow to subgroup the patients according to concomitant MTX. However, the MTX dose remained stable, and the logistic regression did not identify the co-medication as a factor influencing the possibility to stop GCs. Long disease duration and previous treatment failures probably caused the steroid sparing effect of tofacitinib to be underestimated. Still, 30% of patients discontinuing the GCs after only 3 months is an impressive result.

The strength of the study lies in its prospective nature and interventional design that allowed the GCs dose reduction schedule to be predefined to make the outcome homogeneous.

In conclusion, in our cohort of patients with long-standing RA treated with tofacitinib, the discontinuation of glucocorticoids was achievable in up to 30% of patients. The results of the study should encourage rheumatologists to consider GCs tapering and withdrawal a possible goal in the daily management of RA patients long-term treated with oral GC.

### Supplementary Information

Supplementary Table 1.

## Data Availability

The datasets analysed for this study are available from the corresponding author on reasonable request.
