# Supplementary material for: Tapering and discontinuation of glucocorticoids in patients with rheumatoid arthritis treated with tofacitinib
Source: Sci Rep. 2023 Sep 20;13:15537. doi: 10.1038/s41598-023-42371-z (PMC10511736; doi:10.1038/s41598-023-42371-z)
Supplement: Supplementary file 1 — Supplementary Table 1. [file 41598_2023_42371_MOESM1_ESM.docx]

| **baseline** | **week 4** | **week 6** | **week 8** | **week 10** | **week 12** |
| --- | --- | --- | --- | --- | --- |
| 12,5 mg/day | 10 mg/day | 7.5 mg/day | 5 mg/day | 2.5 mg/day | stop |
| 10 mg/day | 7.5 mg/day | 5 mg/day | 2.5 mg/day | 2.5 mg/eod | stop |
| 7.5 mg/day | 5 mg/day | 2.5 mg/day | 2.5 mg/eod | 2.5 mg/eod | stop |
| 5 mg/day | 2.5 mg/day | 2.5 mg/day | 2.5 mg/eod | 2.5 mg/eod | stop |

**Supplementary Table 1.** Schedule of glucocorticoid tapering according to baseline dose.

eod = every other day
